# Use of a Small Peptide Fragment as an Inhibitor of Insulin Fibrillation Process: A Study by High and Low Resolution Spectroscopy

**DOI:** 10.1371/journal.pone.0072318

**Published:** 2013-08-29

**Authors:** Victor Banerjee, Rajiv K. Kar, Aritreyee Datta, Krupakar Parthasarathi, Subhrangsu Chatterjee, Kali P. Das, Anirban Bhunia

**Affiliations:** 1 Department of Chemistry, Bose Institute, Kolkata, India; 2 Biomolecular NMR and Drug Design Laboratory, Department of Biophysics, Bose Institute, Kolkata, India; 3 Department of Microbiology, National University of Singapore, Singapore, Singapore; Russian Academy of Sciences, Institute for Biological Instrumentation, Russian Federation

## Abstract

A non-toxic, nine residue peptide, NIVNVSLVK is shown to interfere with insulin fibrillation by various biophysical methods. Insulin undergoes conformational changes under certain stress conditions leading to amyloid fibrils. Fibrillation of insulin poses a problem in its long-term storage, reducing its efficacy in treating type II diabetes. The dissociation of insulin oligomer to monomer is the key step for the onset of fibrillation. The time course of insulin fibrillation at 62°C using Thioflavin T fluorescence shows an increase in the lag time from 120 min without peptide to 236 min with peptide. Transmission electron micrographs show branched insulin fibrils in its absence and less inter-fibril association in its presence. Upon incubation at 62°C and pH 2.6, insulin lost some α-helical structure as seen by Fourier transformed infra-red spectroscopy (FT-IR), but if the peptide is added, secondary structure is almost fully maintained for 3 h, though lost partially at 4 h. FT-IR spectroscopy also shows that insulin forms the cross beta structure indicative of fibrils beyond 2 h, but in the presence of the peptide, α-helix retention is seen till 4 h. Both size exclusion chromatography and dynamic light scattering show that insulin primarily exists as trimer, whose conversion to a monomer is resisted by the peptide. Saturation transfer difference nuclear magnetic resonance confirms that the hydrophobic residues in the peptide are in close contact with an insulin hydrophobic groove. Molecular dynamics simulations in conjunction with principal component analyses reveal how the peptide interrupts insulin fibrillation. *In vitro* hemolytic activity of the peptide showed insignificant cytotoxicity against HT1080 cells. The insulin aggregation is probed due to the inter play of two key residues, Phe^B24^ and Tyr^B26^ monitored from molecular dynamics simulations studies. Further new peptide based leads may be developed from this nine residue peptide.

## Introduction

Protein fibrillation is one of the important physiological processes that have been linked to development of many pathological conditions, affecting the quality of life for mankind [1]. Alzheimer's disease, Prion-associated encephalopathies, Huntington's disease, Type II diabetes, Parkinson's disease and many more diseases have been linked to protein fibrillation [2], [3]. During the fibrillation process globular proteins undergo structural perturbation followed by a series of structural transitions leading to the formation of insoluble fibrils, containing β-cross sheet like structure [4]. Excessive deposition/accumulation of stable, ordered and amorphous protein aggregates are collectively called amyloid fibrils [5]. Deposition of excessive stable, ordered and amorphous protein aggregates in organs and tissues lead to crucial biological dysfunctions and deleterious pathological symptoms [6]. Researches till date have explored the fact that despite the diversity in sequence homology, the protein fibrils share similar formation pathways and similar morphologies. However, the exact biochemical/biophysical pathways and mechanism of protein fibrillation are yet to be fully understood [7], [8].

Insulin, a 51-residue protein hormone, is central for regulation of blood-glucose level [9]. Due to its propensity to undergo stress induced conformational changes leading to various aggregated forms including amyloid fibrils, it has become the subject of intense study not only as a model fibril forming system but also as the insulin instability issue that reduces the efficacy of insulin therapy against type II diabetes [10]–[12]. The pathological fingerprints of fibrillar insulin have been reported with amyloid deposits in patients with diabetes and in normal ageing [13]–[15]. Once such a deposition takes place, it is mostly irreversible, and may lead to deleterious physiological conditions [16]. It has been reported that soluble oligomers formed at the pre-fibrillar state produces most toxic effects [17]–[19]. Thus, research aiming to delay or inhibit the fibrillation process of insulin can prove to be of great therapeutic value.

Several reports were aimed at developing therapeutic agents to inhibit or slow down the amyloid formation [20]–[21]. These agents routed via natural source or synthetic pathways are generally termed as amyloid inhibitors. The idea behind such anti-amyloid agent is to inhibit the creation of amyloidogenic aggregate and/to arrest the amyloid construction. Insulin consists of two polypeptide chains (Chain A –21 residues; Chain B –30 residues) held together by two disulphide bonds. The solution state of insulin contains a mixture of different oligomeric species including hexamer, tetramer, dimer and monomer [22]. Chain A consists of two helical sections: A2–A8 and A13–A20, whereas Chain B includes a region of extended structure B1–B8, helical section B9–B19, a turn B20–B23 and a terminal extended β-strand B24–B28 [9]. Dissociation of insulin oligomer into monomer facilitates the fibrillation process [10]–[11]. The existence of the conformational intermediates that serve as the precursor for formation of the active nuclei, ultimately culminating into the fibrils is also crucial. Recent work from our laboratory has shown that trifluoroethanol a small molecule could delay the fibrillation of insulin by interfering with the oligomer to monomer dissociation step [23]. Based on these findings, it appears logical to target such steps in order to prevent or slow down the fibrillation process.

Peptides consisting of natural amino acids can be considered as advantageous, as they are devoid of many drawbacks and thus preferred as therapeutics. In the present study we are reporting a nine-residue peptide, NK9 (NIVNVSLVK), which delays the fibrillation process of insulin even in sub-stoichiometric ratio. NK9 is adopted from the middle of the SARS corona virus E-protein primary sequence [24], initially chosen serendipitously. Generally, the polypeptide fragments that have property of self-recognition are important for design of peptide based anti-amyloidogenic agents [25]. The factors, like peptide length and selectivity with respect to L-or D-analogues of peptides, are more crucial according to many groups [26]–[30]. Peptides constituting hydrophobic residues are known to execute inhibition of oligomerization in several biologically important proteins [31]–[33]. NK9 has beta-sheet breaker-hydrophobic residues, like Ile, Val and Leu. The present study helps us to understand the probable mechanism of action for delaying of fibrillation process, where NK9 interferes with the nucleation phase of insulin to decrease the toxicity of the overall process. There are reports that showed different peptides are involved in delaying and/or inhibition of fibrillation process in proteins without giving any significant experimental evidence or emphasis over the cause of the phenomena [27]–[29]. The experimental evidence that NK9 binds with insulin molecule is derived with the help of fluorescence anisotropy experiment. The molecular interaction of peptide with insulin was monitored by means of nuclear magnetic resonance (NMR) spectroscopy and modeled using molecular dynamics simulation [34]–[35] and validated using saturation transfer difference (STD) NMR experiment [36]–[37]. The study helps us understanding the interaction of the peptide with insulin and the underlying mechanism for delaying of fibrillation process.

## Results and Discussion

### General strategies for prevention of insulin fibrillation

One of the early strategies was to increase the stability of insulin by suitable formulation for long-term storage [38]. Since the fibrillation proceeds via dissociation of insulin oligomers into the monomers, strategies for prevention of insulin fibrillation also included among other things promotion of self-association to prevent dissociation into monomer. This was the rationale behind the use of metal ions like Zn^2+^ or Ca^2+^ to stabilize the hexameric structure [39]–[40]. Another approach to stabilize insulin was to promote the native hydrophobic interactions and/or to block unwanted interactions. Polyhydroxy compounds such as carbohydrates and glycerol prohibited water penetration into the hydrophobic core promoting native hydrophobic interactions [41]–[42].

Amphiphilic block co-polymers of polyhydroxy compounds, cyclodextrin and lecithin reduced unwanted hydrophobic interactions by engaging the exposed hydrophobic groups and thus acted as amyloid inhibitors [43]–[44]. Recent studies have explored small molecules such as benzofuranone derivatives and quercetin interfered with the dissociation of the oligomeric insulin to monomer and also destabilized preformed insulin fibril [45]–[46]. Recent studies from our laboratory also showed that trifluoroethanol (TFE) on binding with insulin trimer prevented the appearance of soluble oligomers, believed to be the most toxic product of fibrillation [23].

Use of small peptides for delaying insulin fibrillation has also been reported recently, although molecular details of the interaction of the peptide with insulin remain elusive [47]. We have chosen this 9 residue peptide NK9, which also slows down the insulin fibrillation process. We made a detailed spectroscopic characterization of the fibrillation process of insulin as a function of time and also attempted to get molecular details of the interaction of the peptide with insulin to understand the mechanism of delaying the fibril formation.

### Monitoring of insulin fibrillation kinetics in presence of NK9 via Thioflavin T (ThT) fluorescence

**Figure 1:**
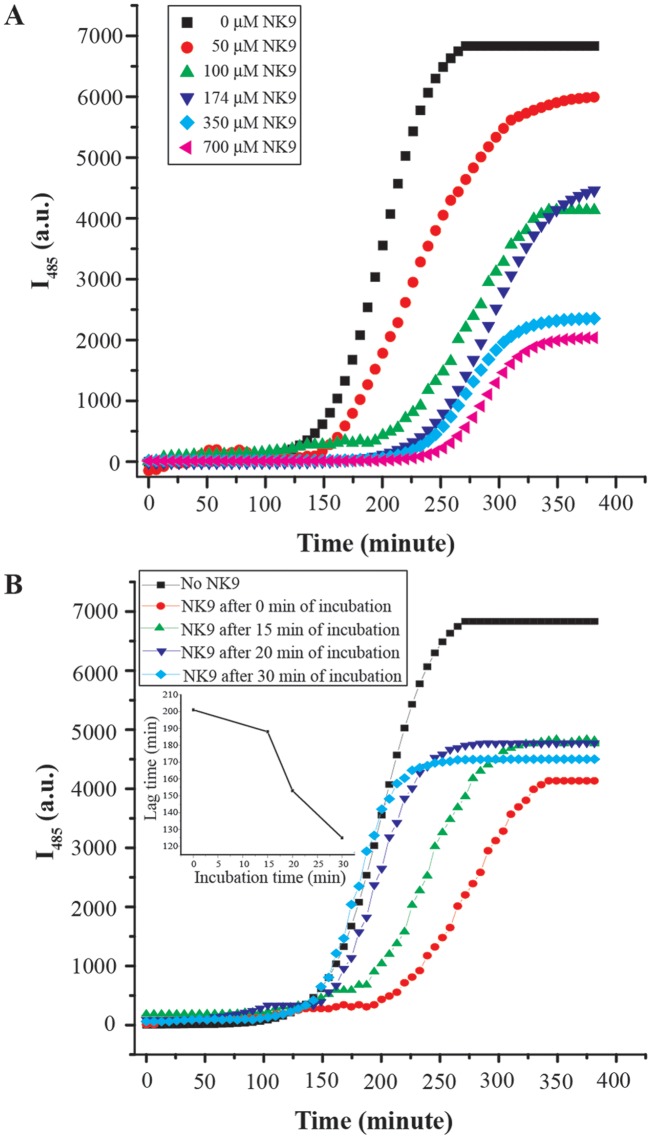
Time course of insulin fibrillation in the presence and absence of NK9, monitored using ThT fluorescence. (A) Temporal evolution of the ThT fluorescence intensity during incubation of insulin (350 μM) in fibrillation condition (pH 2.6, 62°C) in presence and absence of NK9 at different concentrations. ThT curves of insulin in respective NK9 concentrations are mentioned in the figure. (B) Effect of NK9 on fibrillation kinetics of insulin. NK9 (100 μM) was added to the buffer solutions (pH 2.6) of insulin (350 μM) at 62°C at 0, 15, 20 and 30 min of incubation. Molar ratio of NK9 to insulin is 0.28∶1. Inset showed a plot of lag time of insulin fibrillation in presence of 100 μM NK9 against incubation time.

Thioflavin T (ThT) is a dye that preferentially binds to amyloid-like fibrils thereby causing substantial enhancement in its fluorescence emission intensity [48]. Time course of insulin fibrillation in the presence and absence of NK9 was monitored using ThT fluorescence (Figure 1A). Fluorescence measurements were carried out for 7 hr at 62°C. As usual, the ThT fluorescence remains negligible for a considerable period of time (lag time) followed by sharp increase, which eventually leveled off. The lag time for insulin fibrillation is 120 min. NK9 delayed the fibrillation process of insulin in a concentration dependant manner. Even 50 μM concentration of NK9, where molar ratio of insulin to NK9 is 1∶0.14, showed the delaying effect in fibrillation process. At 175 μM concentration of NK9 (1∶0.5 molar ratio), the lag time of fibrillation process was 236 minutes. The lag time increased as NK9 concentration increased (Table S1). However, the lag time remained unchanged after a point in NK9 concentration. Figure 1 also depicts the difference in binding of ThT to insulin fibrils in the absence and presence of NK9 as evident from the difference in the final fluorescence emission intensity values.

In order to understand at what stage NK9 interferes with the insulin fibrillation process, we added NK9 to the incubating insulin solution at 0, 15, 20 and 30 min and studied the effect on the kinetics of fibrillation through monitoring of ThT fluorescence (Figure 1B). We found that maximum lag time (200 min) was observed when NK9 was added at the beginning of the incubation. Nevertheless, when NK9 was added after 15 min of incubation the lag time decreased to ∼190 min. It became 125 min when the peptide was added after 30 min of incubation and this is almost identical with that of insulin without the peptide (Figure 1B). At 30 min addition of NK9, the ThT intensity kinetic profile became very similar to that with insulin alone but the saturation intensity level was lower. In fact, the saturation intensity level of ThT fluorescence was affected very little when the NK9 was added. Thus, maximum protection against fibrillation was obtained when NK9 was present in the system at the time of thermal incubation. This implies that NK9 starts interfering at the initial stages of the nucleation process. This initial stage of nucleation in the absence of NK9 seems to be over in 30 min incubation. Addition of NK9 after 30 min thus did not affect the fibrillation kinetics, but affected only the texture of the fibril possibly by interacting with post nucleation structural intermediates.

### Fibrillation morphology is different for insulin with NK9

**Figure 2:**
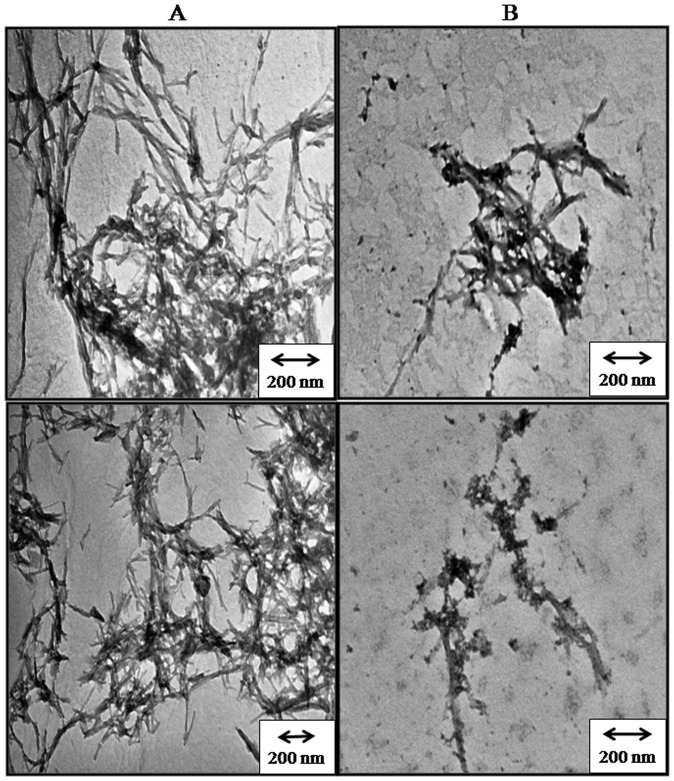
Negative-staining transmission electron micrographs: (A) Bovine insulin and (B) Bovine insulin in presence of NK9. Molar ratio of NK9 to insulin is 1∶1. Insulin fibrils were grown at pH 2.6 and temperature 62°C for 8 hours. Scale bars ∼200 nm.

The difference in binding behavior of ThT with insulin is attributed to the change in fibril morphology of insulin in the absence as well as in the presence of NK9 (Figure 1A). This fact was confirmed by electron micrograph images of insulin fibrils in presence and absence of NK9 (Figure 2). TEM data shows that insulin fibrils in absence of NK9 are branched and those in presence of NK9 are less associated (Figure 2). Nevertheless, the apparent rate constant for fibril growth phase remained almost the same in every case. This indicates that NK9 only delayed the association process in the pre-fibrillar nucleation phase and it does not kinetically affect the post nucleation process leading to fibril growth, although it does have an impact on fibril morphology.

### Circular dichroism spectral changes of insulin during fibrillation in presence of NK9

**Figure 3:**
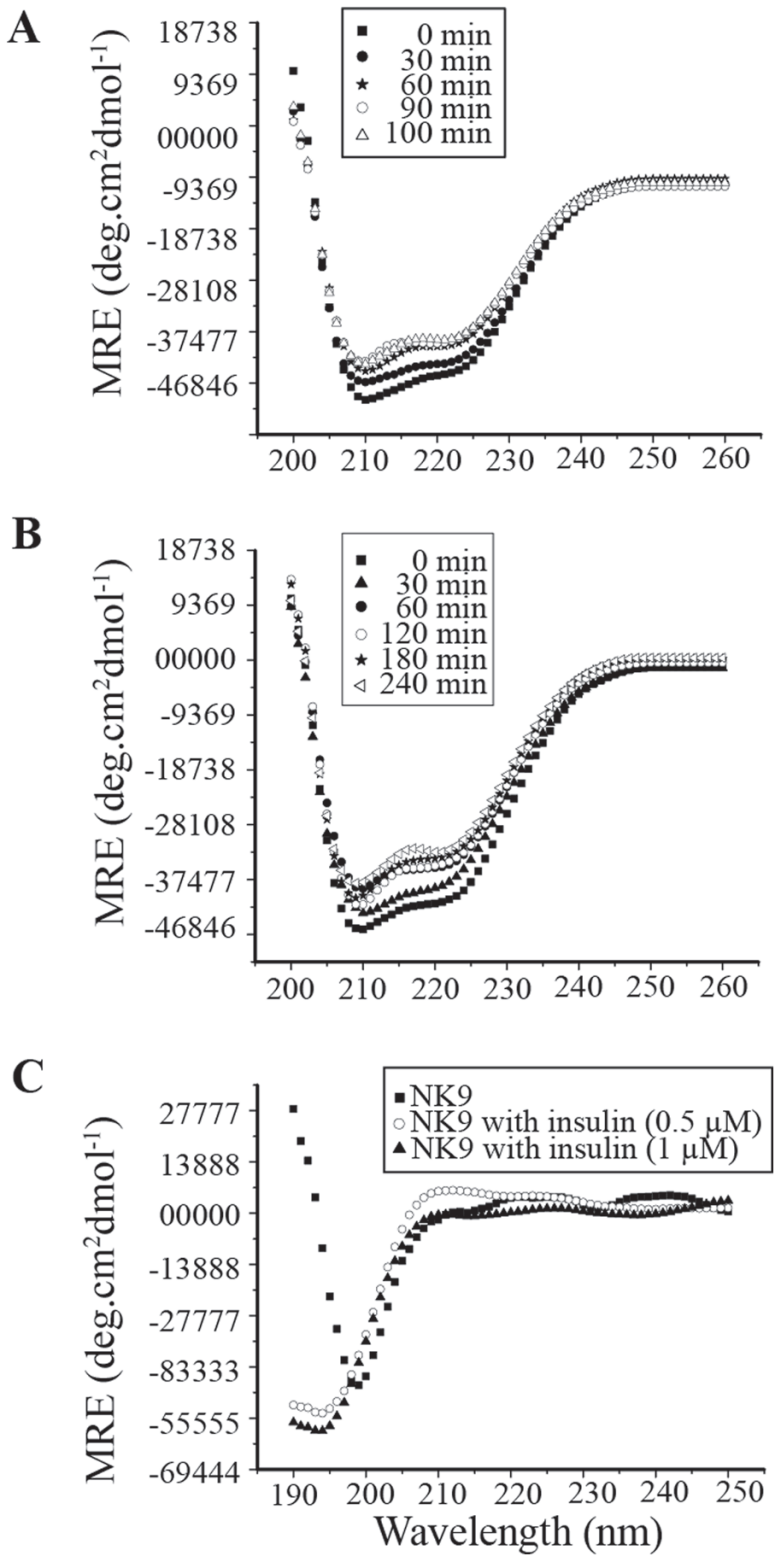
Effect of NK9 binding over secondary structure of insulin during insulin fibrillation monitored by circular dichroism. Selected buffer subtracted far-UV CD spectra of (A) Insulin (B) Insulin in presence of NK9 (NK9 spectra at these selected time points were subtracted from insulin NK9 spectra). (C) Far UV-CD spectra of NK9 in presence and absence of insulin (Insulin spectra was subtracted from NK9 insulin spectra). The concentration of insulin and NK9 both were 350 μM (insulin:NK9 = 1∶1). During the measurement the samples were diluted to 50 μM.

A change in the secondary structure of proteins to cross β-sheet structure is the hall mark of fibril formation [49]. We investigated the changes in the secondary structure of insulin in the absence and presence of NK9 with the help of circular dichroism (CD) spectroscopy. Insulin, a helical protein, shows dual minima at 222 nm and 208 nm that are the characteristics of all major helical proteins. Figure 3A and 3B are the Far UV-CD spectra of insulin at different time points of incubation in the absence as well as presence of NK9, respectively. With the increase in the time of incubation, the negative ellipticity values at 222 nm and 208 nm also decrease, which indicates increased order in the secondary structure of insulin. In order to have a quantitative analysis of the change in secondary structure of insulin during fibrillation process, we have de-convoluted the far UV-CD data with the help of CDNN software. After 100 min of incubation, the helix content of insulin decreased to 39% with little increase in β-sheet structure. Whereas, the secondary structure of insulin in presence of NK9, remained almost unchanged for 180 min of incubation. At 240 min of incubation, helix content of insulin in presence of NK9 decreased only to 42%. This implies that NK9 helps insulin to retain its secondary structure for a prolonged period of time. Appearance of insoluble aggregates makes it difficult to continue the CD measurement beyond 100 min of incubation for insulin alone and 240 min of incubation, for insulin in presence of NK9. Nevertheless, NK9 does not have a well-defined structure even in the presence of insulin (Figure 3C).

### Effect of NK9 on the secondary structural changes during insulin fibrillation: FT-IR study

**Figure 4:**
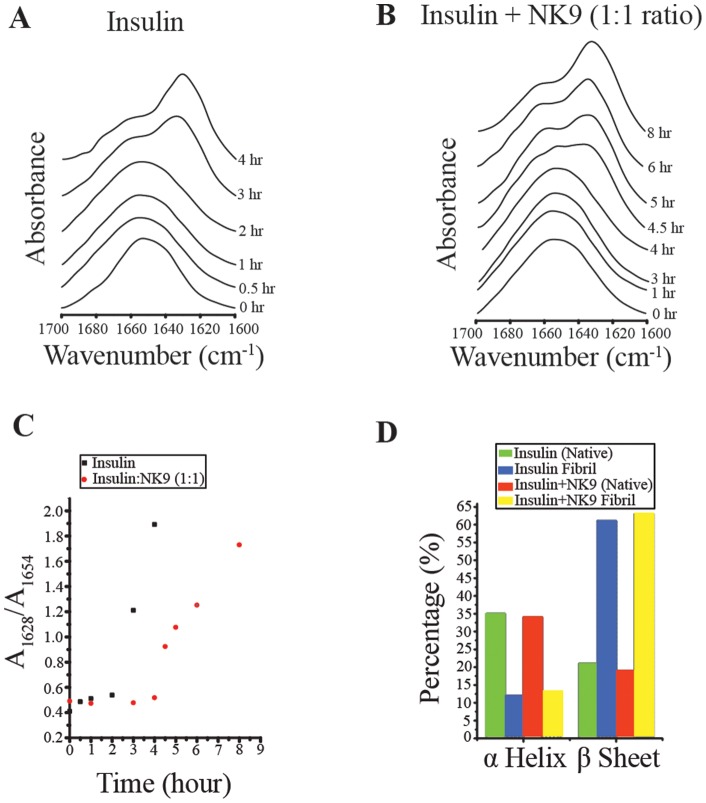
Conformational changes during insulin fibrillation as detected by FT-IR. Time dependent FT-IR spectra of insulin (2.0 mg/ml in D_2_O) in absence (A) and presence (B) of NK9 in 62°C and pD 2.6. Amide-I band were acquired in the 1700–1600 cm^−1^ range at a resolution of 2 cm^−1^. Each spectrum is an average of 32 scans and subtracted from solvent spectrum. Panel C shows the ratio of absorbance of β-sheet peak at 1628 cm^−1^ to helical peak at 1654 cm^−1^. Filled square represents insulin and open circle represents insulin in presence of NK9. Panel D shows percentage of α-helix and β-sheet present in native and fibrillar insulin in presence and absence of NK9. The molar ratio of insulin to NK9 is 1∶1.

FT-IR has been used to study secondary structure of proteins for decades [47], [50]–[53]. It is particularly sensitive to changes of β-sheet content in proteins [43]. Being an α helical protein insulin has a characteristic peak at ∼1654 cm^−1^ in its amide I contour. On conversion into amyloid like fibrils, the peak shifts to 1628 cm^−1^, which is the hallmark of cross β-sheet structure formation [54]. Figure 3 shows FT-IR spectra of insulin measured at various intervals of incubation. Plot of A_1628_/A_1654_ against time of incubation (Figure 4) showed very small changes in the ratio and spectral shape take place for the first 2 h of incubation. The transition involving significant increases in the A_1628_/A_1654_ ratio was observed after 2 h of incubation, and it persisted for 4 h. This is the cause of steady increase in intensity at 1628 cm^−1^, with a concomitant decrease in intensity at 1654 cm^−1^ (Figure 4). This change reflects the formation of cross β sheet structure in insulin at the expense of its helical content. Insulin in presence of NK9 retains its secondary structure for 4 h of incubation (Figure 4). A_1628_/A_1654_ ratio at 4.5 h of incubation showed a much lower value as compared to the ratio in case of insulin alone after 4 h of incubation (Figure 4). This observation indicates that NK9 slowed down the stepwise changes in insulin secondary structure during the process of fibrillation. The amount of helical content left and β-sheet content increased after formation of mature fibril was calculated using the multi curve fitting program of origin 6.0 software (Figure S1). Curve fitting analysis showed that insulin in the presence and absence of NK9 has similar percentages of α-helix and β-sheet content (Figure 4). Although NK9 delays the fibrillation process of insulin, mature fibrils of insulin have ∼60% β-sheet content irrespective of NK9 presence.

### Size exclusion chromatographic study of insulin in presence of NK9 during Fibrillation

**Figure 5:**
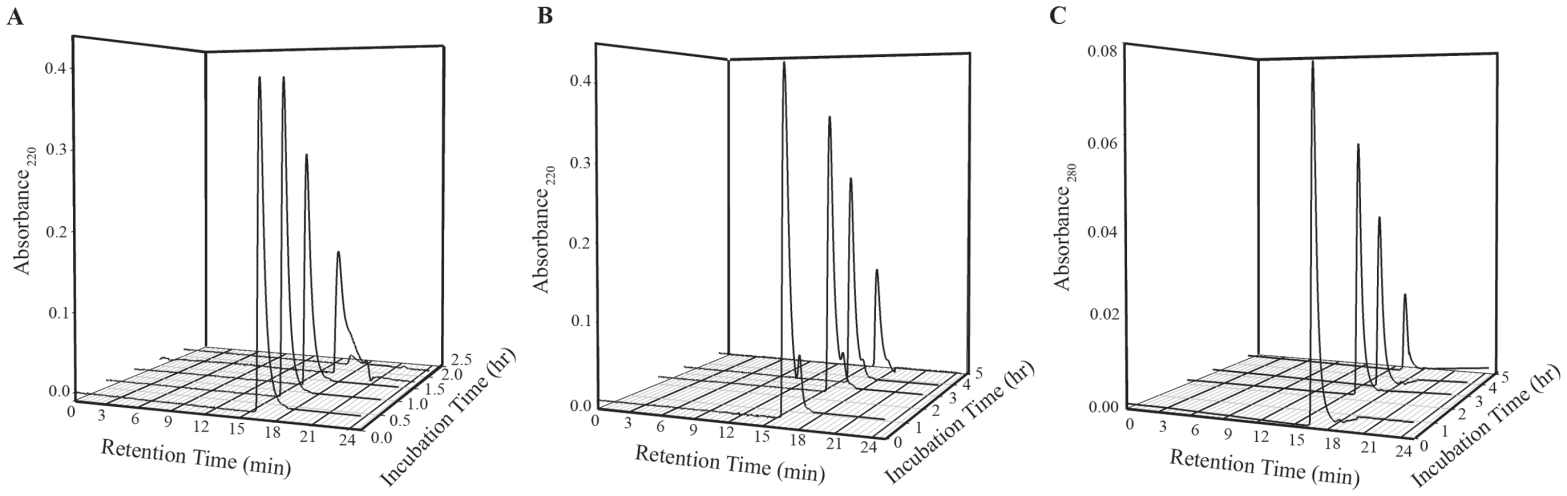
Changes in the association state of insulin in presence and absence of NK9. Absorbance profiles at 220 nm from size exclusion chromatography experiments with insulin (350 μM): (A) Absence and (B) Presence of 350 µM NK9 at different time of incubation. Molar ratio of NK9 to insulin is 1∶1. Panel C shows SEC profile of insulin in presence of NK9 at 280 nm wavelength to distinguish insulin peak from the NK9.

The association states of insulin in the presence and absence of NK9 were determined by the size exclusion chromatography using TSKgel SuperSW2000 HPLC column. The column was pre-calibrated using size exclusion marker proteins β-amylase, ADH, BSA, carbonic anhydrase, lysozyme, and ribonuclease. The calibration data fitted nicely into a linear equation (correlation coefficient, R^2^ = 0.98). Since incubation of insulin samples for fibrillation experiment were done using citrate phosphate buffer of pH 2.6, the HPLC column was equilibrated with this buffer for experiment with insulin. It was confirmed by using BSA and lysozyme that both proteins retained their globular shape as retention time at this acidic pH did not significantly change from those at pH 7.0 (data not shown). Aliquots of insulin solution at different time points of incubation were centrifuged at 4000×g force to remove visible turbidity. Supernatant was loaded on to the HPLC column. Prior incubation of insulin in citrate phosphate buffer showed a retention time of 16.9 ml (Figure 5A). This retention time corresponds to the trimeric structure of insulin that corroborates well with earlier findings by Banga and co-workers [39]. Retention time of insulin remained unchanged for 1.5 hr with concomitant decrease in its absorbance value. The increase in incubation time up to 2 hr, shifted the retention time of insulin to 17.9 min that corresponds to the monomeric insulin. After 2 hr of incubation, fluorescence emission intensity just started to increase (Figure 1) implying that active nucleus of insulin fibrils was formed just after 2 hr of incubation. The size exclusion chromatography data shows that the building block of this active nucleus is a monomer of insulin. This conclusion is in agreement with the literature data as well [55].

Insulin incubated with NK9 (1∶1 molar ratio) showed two distinct retention times, one was at 16.9 min and another one was at 18.1 min. The first peak could be due to Insulin-NK9 complex and the latter for NK9 alone (Figure 5B). Since NK9 does not contain any tryptophan residue there was no peak appeared at 18.1 min when absorbance values were taken at wavelength 280 nm (Figure 5C). This also confirms that the peak appeared at 18.1 min was for NK9 alone. Since NK9 is a short peptide, binding with insulin does not have any significant influence on molecular mass and on the retention time of insulin. Consequently, the retention time of insulin-NK9 complex is identical with that of insulin alone. As incubation time proceeds, the absorbance value at both retention time points decreased. This indicates that the insoluble part of insulin, contain the NK9 peptide. Since there was not much change in retention time of insulin-NK9 complex during 5 hr of incubation, it is understood that NK9 stabilizes the associated trimeric state of insulin.

### Association state of insulin during fibrillation in presence NK9

**Figure 6:**
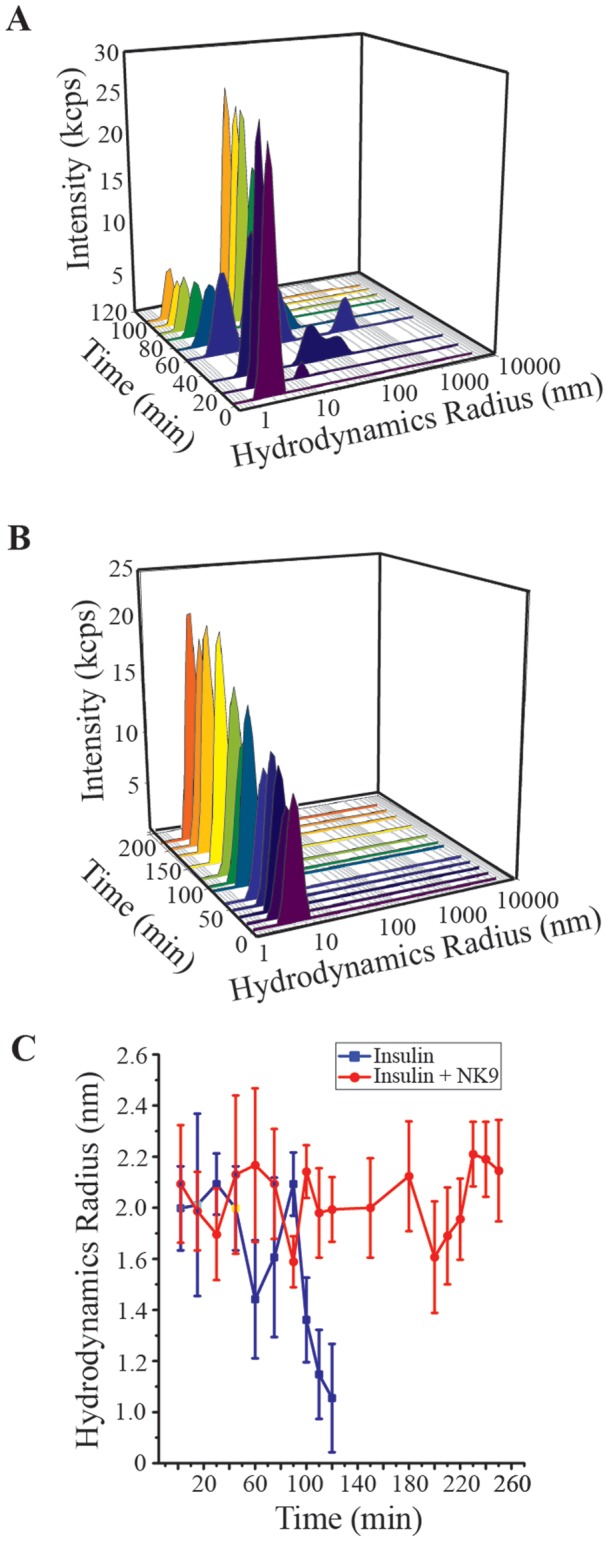
Intensity particle size distribution spectra of bovine insulin: (A) Absence and (B) Presence of 350 µM NK9 (NK9 : insulin = 1 ∶**1).** Each spectrum is an average of 36 scans. The figure shows the results of dynamic light scattering analysis as a function of incubation time. Panel C shows hydrodynamic radius of 350 μM insulin, in presence and absence of NK9 at different times of incubation at fibrillation inducing environment. Filled square represents insulin and open circle represents insulin in presence of NK9. Each DLS measurement is an average of 36 scans and the error bar represents the standard deviation of those 36 measurements.

Dynamic light scattering experiment showed that the hydrodynamic radius of insulin in the presence and absence of NK9 was 2.1 and 2.0 nm, respectively, that corresponds to the trimeric state of insulin (Figure 6). After 30 min of incubation, insulin associated to form a large oligomer of 24 nm in size along with its trimeric association state (Figure 6A). As the time of incubation increased, population of 24 nm sized oligomer increased and retained up to 120 min of incubation. Though, the intensity scattered by the higher oligomer is more than the trimeric association state of insulin, the percentage of trimeric insulin is much more than that of the higher oligomer. This observation is also evident from our size exclusion chromatography data. After 100 min of incubation, insulin trimer disintegrates and forms insulin monomer of 1.3 nm (Figure 6). This is believed to be the building block of active nucleus of the fibrillation process [56]. Insulin in presence of NK9 shows hydrodynamic radii of 2.1 nm indicating the trimeric association state. This 0.1 nm increase is within experimental error, so we cannot say that this increment is due to binding with a 9 residue peptide. However, subtle change in conformation of insulin bound with NK9 is reflected due to the increase in scatting intensity as the incubation time increases (Figure 6B). Insulin in the presence of NK9 retained its trimeric state up to 240 minutes of incubation (Figure 6C). However, after 240 min of incubation, insulin started to form visible precipitates and renders the DLS experiment unsuitable to be conducted for further time points.

### Fluorescence anisotropy (FA) analysis of the interaction of NK9 with insulin

**Figure 7:**
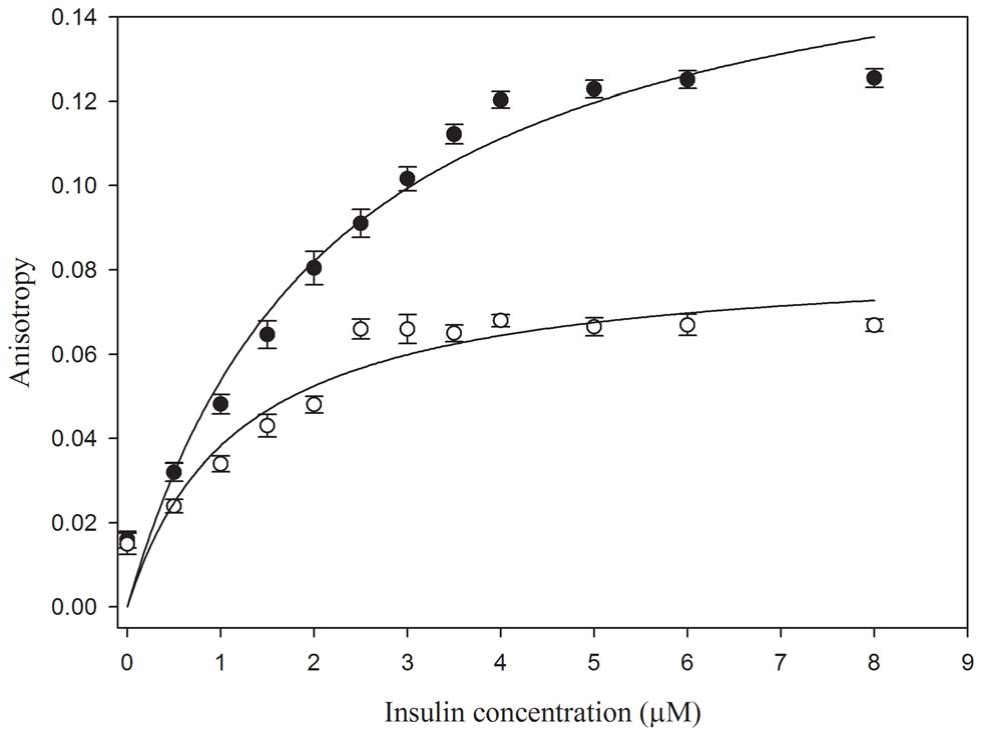
Fluorescence anisotropy titration of insulin. Anisotropy of 10 µM FITC tagged NK9 is plotted as a function of increasing insulin concentration at 25 and 37°C temperature. The error bars represents the standard deviation of 3 measurements.

Fluorescence anisotropy (FA) is a useful technique to study the binding interaction of a fluorescently labeled ligand with proteins. Theoretically, as the interaction between the ligand and the protein increases, rotational diffusion of the ligand decreases, that results in increase in anisotropy [57]. In Figure 7 as the concentration of insulin increases, the anisotropy also increases rapidly in the initial part of the curve. Thus, in the beginning, the added protein was immediately bound to NK9. The increase in anisotropy became smaller and finally reached a plateau. The plateau reflects anisotropy of the saturated insulin NK9 complex. This experiment was carried out at two different temperatures (25°C and 37°C) to validate the binding of NK9 with insulin at higher temperature. Anisotropy experiment at 25°C shows the plateau beyond an insulin concentration of 4 µM, which implies almost 2∶1 binding of NK9 to insulin. Whereas, anisotropy experiment at 37°C showed plateau beyond 2 μM concentration of insulin implying almost 4∶1 binding of NK9 to insulin. Binding curves were obtained with apparent K_d_ values of 2.2 μM and 1.2 μM for binding to insulin at 25°C and 37°C, respectively. Gibbs free energy change for this association process was found to be −32.2 and −35.1 KJ.mol^−1^ at 25°C and 37°C respectively. This observation clearly indicates that the rate of insulin-NK9 complex formation increases at elevated temperatures.

### Identification of probable binding site of NK9 with insulin by STD NMR and cluster analysis

**Figure 8:**
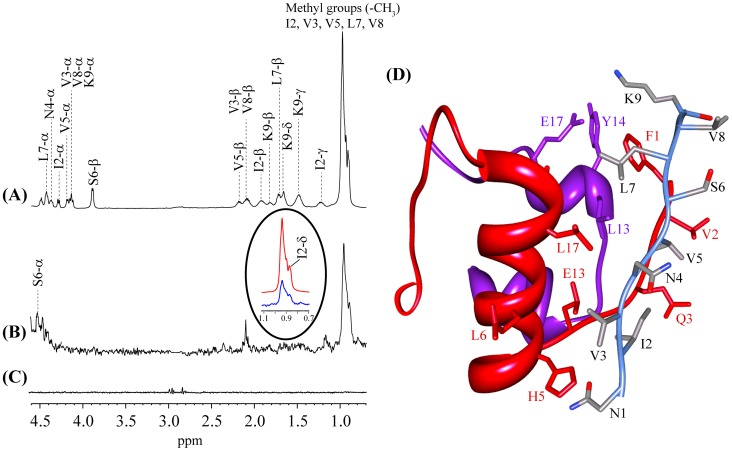
STD NMR Spectrum. (A) Reference spectrum of NK9 in presence of insulin at a molar ratio of insulin:NK9 = 1∶100. (B) STD NMR spectrum of same sample showing the resonance transfer within the binding epitope of insulin. The experiments were performed at 298 K with 2 sec of saturation time (on resonance  = 7.3 ppm; off resonance  = 40 ppm). (C) STD spectrum of NK9 alone (on resonance  = 7.3 ppm; off resonance  = 40 ppm). (D) The docking model of insulin-NK9 complex correlated of the STD NMR data.

To obtain the structural insight of NK9-insulin complex, transferred NOESY experiment was performed (Figure S1). The peptide in absence and presence of insulin was predominantly characterized by intra residue and sequential NOEs between backbone proton and side chain proton resonances (Figure S1), clearly indicating that the peptide is highly flexible in solution state and does not adopt any folded conformation in presence of insulin. This result is in very good agreement with our CD data of NK9 in the context of insulin (Figure 3C). To understand the localization of NK9 in insulin we employed one dimensional proton STD NMR which is a powerful and very specific technique to identify the epitope of a ligand is in close proximity with the receptor [37]. Briefly, selective saturation to the receptor is transferred to the bound ligand via spin diffusion, without affecting the ligand signal alone. Therefore, from the difference spectrum (off resonance – on resonance) we obtain the distance information or the epitope of the ligand bound to the macromolecule. Excess of ligand compared to its receptor is used in the STD experiments to achieve effective magnetization transfer from the receptor to the ligand at its bound state (Figure 8). The strong STD effect was observed for the methyl groups of NK9 in presence of insulin (Figure 8B) but there was no STD effect in the absence of insulin (Figure 8C). Although it is difficult to pin point the aliphatic hydrophobic amino acid residue due to significant overlap of the methyl group signals, it could be from any or all of the residues from I2, V3, V5, L7 and V8 closely interacting with insulin. The I2γ and C^β^Hs of V3/V5/V8 of NK9 show moderate STD effects (Figure 8B). The C^α^Hs of NK9 are very close in proximity to the water signal. Interestingly, the C^α^H of S6 and L7 showed very weak STD signals (Figure 8B). Taken together, STD data qualitatively suggest a close association of hydrophobic amino acid residues with insulin (Figure 8B).

Molecular modeling is used to get structural insights of insulin-NK9 complex. Figure 8D shows the docked model, where NK9 is found to be in close proximity within the molecular patch between N-ter and helical portion of chain B. The key residues of insulin found to be involved in contact with NK9 are Phe^B1^, Val^B2^, Gln^B3^, Leu^B6^, His^B10^ and Leu^B17^. In contrast, only the few residues of chain A, namely Leu^A13^, Tyr^A14^ and Glu^A17^ showed proximity to the residues of NK9. The docking model is in very good agreement with the STD NMR result, where all the methyl groups, I2, V3, V5 and L7 of NK9 are in hydrophobic groove, formed by the aliphatic residues of chain A and chain B of insulin. The N1 residue of NK9 is close to His^B5^, suggesting formation of salt bridges or hydrogen bond. The model also suggests interaction between N4 of NK9 and GlnB3 of insulin by either hydrogen bond or salt bridges and hydrogen bond between K9 of NK9 and Tyr^A14^ of insulin. Probable fibrillation site of insulin and insulin NK9 complex was also identified using BioLuminate software (Figure S2).

### Interaction within insulin-NK9 complex in explicit conditions; trajectory analysis

The physical behavior of the peptide (NK9) with macro-molecule (insulin) during the 100 ns MD simulation was accessed by Root Mean Squared deviation (RMSD), Radius of Gyration (Rg), Root Mean Square fluctuations (RMSF) and Principal Component Analysis (PCA).

**Figure 9:**
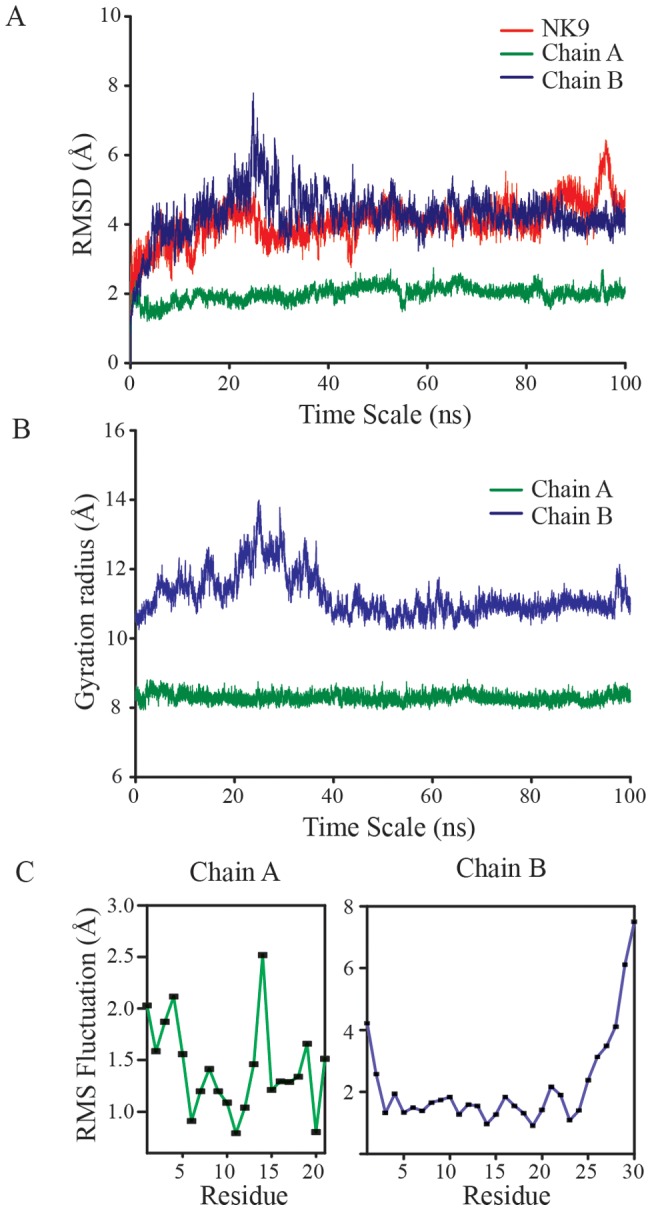
A 100 ns MD simulation results for insulin-NK9 complex. (A) RMSD plot for chain A and B of insulin and NK9. (B) Radius of gyration for chain A and B of insulin. (C) Root mean square fluctuation of individual residues corresponding to chain A and B of insulin. Various color codes are used to distinguish each chain in the plot.

Figure 9A shows all-atom RMSD for individual chains of insulin and NK9. The dynamicity of the complex seems to be stable and unaltered even after 20 ns. The N-terminus of the chain B enforces a wide range of dynamicity. The docked model designates the location of NK9 binding just in between the N-ter and helical region of chain A. The fluctuation in chain B is tuned and regulated by NK9, restricting the fibril formation. The RMSD plot for chain A is found to be unaffected in the simulation course. There is no observation regarding the loss of secondary structure in any chain of insulin that corresponds to the stability of whole structure. The Rg as shown in Figure 9B, correlates nicely with the RMSD data points. The minute increase in Rg is genuine and can be attributed to the absence of crystal packing in the micro-canonical ensemble of simulation, but the large increment can be correlated to the interaction with NK9. The disulfide bridges of insulin are found to be well conserved at low pH simulation, indicating one of the reasons for the stability regarding rms deviation and gyration radius of chain A. The results discussed for RMSD and Rg plot can be more clearly explained with the help of RMSF fluctuations for both chain A and B (Figure 9C). The fluctuation with respect to the Cα atoms of chain A residues are small except for Tyr^A14^, which is found to be in contact with Lys^9^ of NK9. The observed mean fluctuation of 2.5 Å is attributed to cation-π type interaction between these two residues. The RMSF for chain B shows a major fluctuation corresponding to Lys^B29^ and Ala^B30^ residues which are in the N-terminal region.

**Figure 10:**
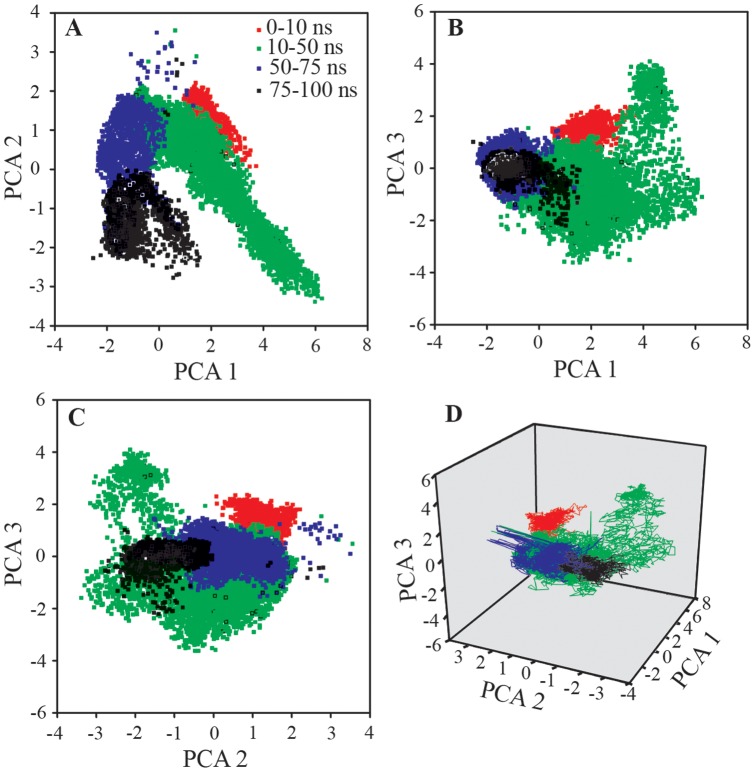
Principal component analysis (PCA) considering the essential dynamics of prime three eigenvectors viz. PCA1, PCA2 and PCA3.

PCA is one of the important tools to describe the stochastic movement of macromolecular system. All the conformations sampled for simulation, were analyzed in terms of linear relationships between atomic motions using the first three prime eigenvectors. The correlation scatter plots link the motions of ‘related’ fluctuations with those of ‘total’ fluctuations in the system, and are directly related to their biophysical properties. Figure 10 shows the projections of first three principal components. All scatter data-points were categorized by color codes to give an approximation of the path followed by insulin-NK9 complex in the simulation time course. The scatter point corresponding to 10–50 ns shows the time portion of simulation where the complex adopted many of the conformational changes for finding favorable interaction between chain B and NK9. Interestingly, the atomic fluctuations seem to have converged in the next phase of analysis (50–75 ns) and were almost conserved as for global minima in the phase 75–100 ns. PCA helps to concluding that the structural conformations as obtained beyond 75 ns is more close to stable conformation at low pH conditions.

REMD simulation gives certain insight based upon the temperature platform. The replicas, which actually mimic the amyloid form, are checked at low pH 2.0 and at the temperatures 330 K and 335 K. The trajectories were found to be stable for chain B. Interestingly, the replica exchange study confirms that the conformational switch based on temperature variation does not cross the energy barrier to reach the form defined as amyloid plaque. This is the reason the replicas do not show disordered deviation plots (Figure S3).

### *In vitro* hemolysis and cytotoxicity assay of NK9 peptide

**Figure 11:**
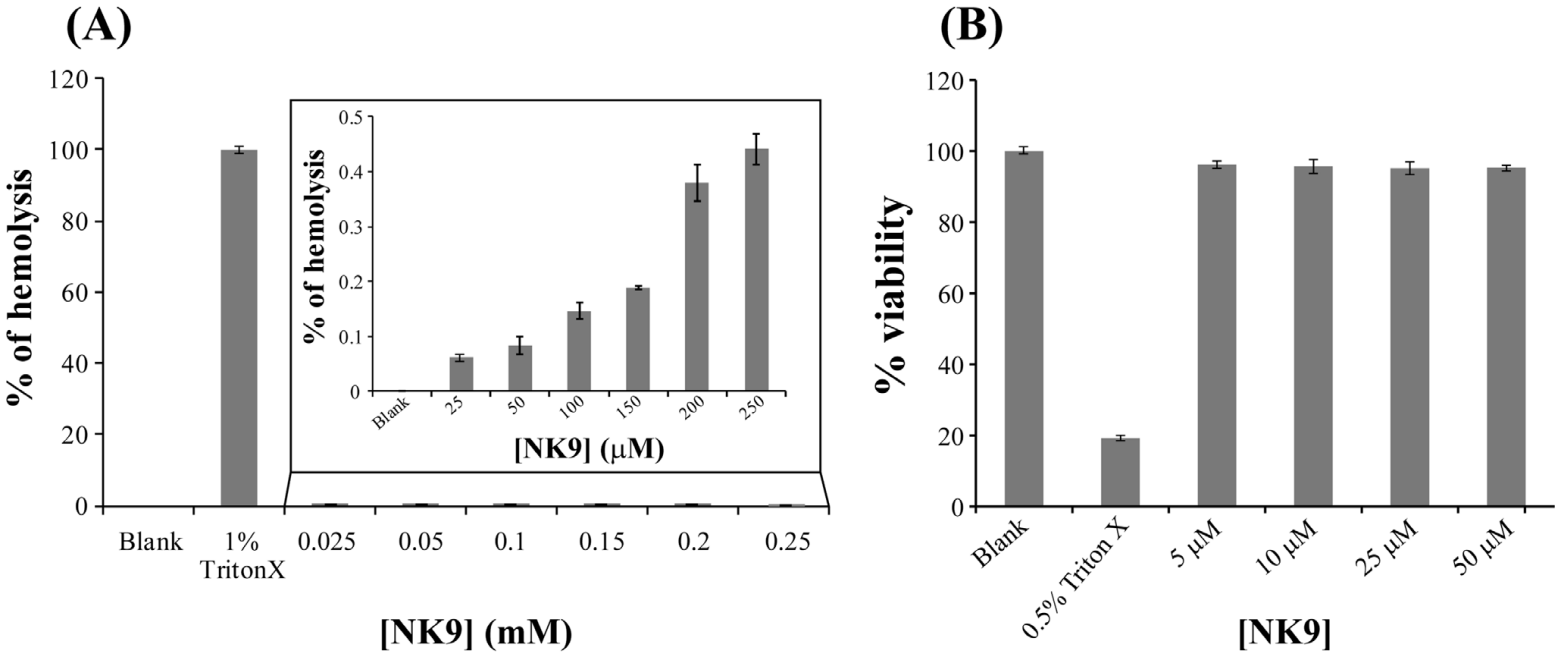
Hemolytic and cytotoxity assay of NK9 peptide. (A) Percentage hemolysis of NK9 peptide on human red blood cells exhibiting less than 0.5% hemolysis for different concentrations (25, 50, 100, 150, 200 and 250 µM) of peptide been tested. Blank corresponds to untreated red blood cells (without peptide). The positive control for this experiment was 1% Triton X 100. (B) Percentage viability of HT1080 cells upon treatment with varying concentrations (5, 10, 25 and 50 µM) of NK9 peptide showing less than 5% cytotoxicity in each case, against untreated cells taken as blank. The positive control for this experiment was 0.5% Triton X 100.

*In vitro* hemolysis assay evaluates hemoglobin release in the plasma as an indicator of red blood cell lysis following exposure to the drug or agent under consideration. It is an accurate and sensitive method for predicting hemolytic activity of a drug. The study of hemolytic activity of NK9 peptide showed less than 0.5% hemolysis for the various concentrations of peptide assayed up to a final concentration of 250 µM (Figure 11). Percentage hemolysis was measured with respect to 1% Triton X 100 sample taken as a control, which was considered to exhibit 100% hemolysis. The experiment was conducted in three biological replicates and similar results were obtained in each case. Cytotoxicity assay of NK9 peptide on HT1080 cell line was also performed for an analysis of its effects on live cells post treatment. No significant (less than 5%) cytotoxicity was observed towards HT1080 cell line for the different concentrations being tested going up to a final concentration of 50 µM. 0.5% Triton X 100 was used as a control, which exhibited only 20% viability. Cytotoxicity was measured by calculating the percentage of viable cells at each concentration of NK9 peptide relative to the control sample without peptide, which was considered to have 100% viability. The experiment was repeated in 4 replicates and yielded similar results. These data clearly indicate that the peptide, NK9 is non-toxic and the lead compound(s) from this peptide sequence can pave way to develop of new generation anti-diabetic drugs.

## Materials and Methods

The protein, insulin and pure peptide, NK9 studied in this project were purchased from Sigma (St. Louis, MO) and GL Biochem (Shanghai, China), respectively. The molecular weight of the peptide was confirmed by ESI mass spectrometry.

### Human samples

Human blood from healthy volunteers was collected using approved protocol from the Human Ethics committee, Bose Institute, Kolkata. Blood was drawn by experienced clinical technician after obtaining written consent from the volunteers and the hemolytic assay was performed with prior approval of the protocol from the said committee.

### Preparation of samples

Bovine insulin was dissolved in 50 mM citrate phosphate buffer of pH 2.6. The concentration of sample solution was determined by measuring the absorbance at 276 nm using extinction co-efficient of insulin as 0.91 (mg/ml) ^−1^.cm^−1^ [56]. The buffer solutions were degassed for 15 min with vigorous stirring and filtering through 0.22 µm disk membrane immediately before use. The degassing process was done with an objective to avoid any interference of dust and micro bubbles in the experiments. Thioflavin T (ThT) was dissolved in mili Q water and its concentration was determined from its absorbance at 412 nm using the molar extinction co-efficient of 36,000 M^−1^ cm^−1^ [57].

### Kinetics of fibrillation process

ThT fluorescence of each sample was monitored in 2 mm path length cuvette incubating at 62°C temperature in Hitachi F 4500 spectrofluorometer. Excitation and emission wavelengths were set for measurement as 450 nm and 485 nm, respectively. Insulin concentration in the cuvette was 2 mg/ml and that of ThT was 25 µM. In order to have reproducibility of results, all the ThT date are average of three measurements. The kinetic profile was analyzed by curve fitting into Boltzmann equation (a sigmoidal function) using Origin software v.6.0.

### Analysis of secondary structure of insulin during different stages of fibrillation by CD spectroscopy

The far-UV CD spectrum was recorded at 25°C for wavelength range of 200–250 ns on a JASCO-810 Spectropolarimeter using a rectangular cell of 1 mm path length. Insulin solution (2 mg.ml^−1^) at pH 2.6 was incubated in a water bath at 62°C. Aliquots of insulin solution were taken out at different time of incubation and diluted to 0.3 mg.ml^−1^. To eliminate contributions of NK9 in the Insulin NK9 mix system, NK9 also was separately incubated and aliquots were taken out at different time instants of incubation to measure the CD spectra and then subtracted with the Insulin NK9 CD spectra at respective time points. A buffer spectrum was also subtracted with each spectrum to eliminate contribution of the contributions of buffer. The CD results were expressed in terms of Mean Residue Ellipticity (MRE).

### Monitoring of secondary structure of insulin during fibrillation by FT-IR spectroscopy

For FT-IR measurement, incubated insulin solution (50 μl) was taken in microcon filter device fitted with a 3-kDa cut-off membrane and diluted with ∼200 μl heavy water (D_2_O) of pD 2.6. The sample was centrifuged at 4000×g for 8 to 10 min to bring the volume down to ∼50 μl. It was further diluted with ∼200 μl D_2_O and centrifuged again. This deuterium exchange process was repeated for 3–4 times. Finally, the D_2_O exchanged insulin (solution/suspension, 15 μl) was placed between two CaF_2_ windows separated by a 50 μm thick teflon spacer. FT-IR scans were collected in the range of 1600–1700 cm^−1^ at a resolution of 2 cm^−1^ using a Spectrum 100 FT-IR spectrometer (Perkin Elmer). Spectrum of D_2_O at pD 2.6 was collected and subtracted from each sample spectrum. Fourier self-deconvolution was used to resolve overlapping bands in the FT-IR spectrum. Curve fitting of the original amide I contours was performed using Microcal Origin 6.0 multi peak fit programme. Assignment of amide I bands were done according to the literature data [58]. The percentage of each secondary structure was computed to be the fractional area of the corresponding peaks, divided by the sum of the areas of all the peaks in amide I band. Curve fitting of FT-IR data was done with the help of origin 6.0 software using multi curve fit programme.

### Transmission Electron Microscopy (TEM)

Imaging of fibril samples was done by using a transmission electron microscope (Technai G2 sprit BioTWIN, FEI) with an acceleration voltage of 80 kV. Aliquots (10 μl) of insulin solution were placed on the copper grid coated with carbon film (300 meshes, Agar Scientific, Stansted, UK). After 30 s, two drops containing 10 μl of 2% uranyl acetate (Agar Scientific) were placed on the grid. The excess water was removed carefully with filter paper and the grid was left to dry in air.

### Measurement of hydrodynamics radius (RM) of insulin oligomers by DLS

The stock insulin solution was filtered through 0.2 μm disk membrane and then diluted to a final concentration of 2.0 mg/ml. The experiments were performed at pre-adjusted temperature of 62°C, at which fibrillation took place over a time period of 4 h. Size of the insulin in the early stages of the fibrillation were measured by dynamic light scattering (DLS) employing a Zetasizer Nano S (Malvern Instruments). DLS technique measures the time dependent fluctuation in the intensity of scattered light that occurs because of the motion of the particles. The analysis of these fluctuations enables the determination of the translational diffusion coefficients (D) of particles, which can be transformed to a size distribution. The value of D is obtained from the fitting of the experimental values of the time correlation function g_2_(τ) according to the Equation 1.
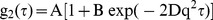
(1)where, τ is the time, A is the baseline of the correlation function, B is the intercept of the correlation function, q  =  (4лn/λ) sin (θ/2)] is the scattering vector, n is the refractive index of the solution, λ is the wavelength of the laser, and θ is the scattering angle. A 4-mW He–Ne laser (633 nm) with a fixed detector angle of θ = 173° was used. The time-dependent autocorrelation function was acquired every 60 s, with ten acquisitions for each run. Each data is an average of three such acquisitions.

### Size exclusion chromatography

Size exclusion chromatography was performed using TSKgel SuperSW2000 column from Tosoh. The column was run on a waters 1525 HPLC system at a flow rate of 0.35 ml.min^−1^ with dual mode detection at 280 and 220 nm. The column was equilibrated with 25 mM citrate phosphate buffer to obtain a stable base line. After that 20 μl of 2.0 mg/ml insulin solutions at different incubation times was first centrifuged and then supernatant of the solution was loaded on the column to see the association state of insulin in presence and absence of NK9. Markers such as: β amylase, ADH, BSA, carbonic anhydrase, lysozyme and ribonuclease were run in 25 mM phosphate buffer pH 7.2. However, these proteins retain their globular shape at low pH condition, which was confirmed by BSA and lysozyme. Both BSA and lysozyme maintained their retention volume at pH 2.6 (data not shown).

### Fluorescence anisotropy measurement

Steady-state anisotropy was recorded with a Hitachi model F-4500 spectrofluorometer equipped with a polarization accessory. Excitation of FITC labeled NK9 was done at 453 nm. The fluorescence anisotropy (A) values were obtained using the expression as of Equation 2.
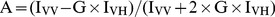
(2)where, I_VV_ and I_VH_ are the vertically and horizontally polarized components of probe (emission wavelength 526 nm) with excitation by vertically polarized light at 453 nm. G is the sensitivity factor of the instrument. The excitation and emission slits were set to 5 nm. The FITC tagged NK9 concentration was 10 μM, whereas insulin concentration was varied between 0.5 to 8 μM. The curve data were fitted using one site binding model of SigmaPlot software v.10.0. according to the Equation 3.
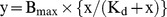
(3)where, B_max_ is the maximum value of anisotropy during the binding process and K_d_ is the dissociation constant of insulin NK9 complex. These experiments were carried out at 25°C and 37°C temperatures.

### NMR experiments

All NMR spectra were recorded on a BRUKER AVANCE III 500 spectrometer, equipped with pulse field gradients. Data acquisition and processing were performed with Topspin software suite (BRUKER). Two-dimensional TOCSY and NOESY spectra of NK9 were acquired in buffer solutions containing 10% D_2_O at pH 2.6 with a peptide concentration of 1 mM. The mixing times for TOCSY and NOESY experiments were set to 80 ms and 400 ms, respectively at 298 K using DSS (2,2- Dimethyl-2-silapentane-5-sulfonate sodium salt) as an internal chemical shift reference. A series of one-dimensional proton NMR spectra were acquired by stepwise additions of various concentrations of insulin from a stock solution of 1 mM. The peptides/insulin complexes were carefully monitored. Two-dimensional transferred NOESY (*tr*NOESY) experiments were carried out at a Peptides/insulin molar ratio of 12∶1 at 298 K. Three different NOESY mixing times; 100, 150 and 200 ms were recorded with 456 increments in t1 and 2 K data points in t2. The spectral width was normally 12 ppm in both dimensions. After 16 dummy scans, 120 scans were recorded per t1 increment. After zero filling in t1, 4K (t2) ×1K (t1) data matrices were obtained. NMR data analysis were carried out using the program SPARKY (T. D. Goddard and D. G. Kneller, University of California, San Francisco).

For saturation transfer difference (STD) NMR experiments, insulin and NK9 were dissolved in a buffer containing 99.9% D_2_O and lyophilized for at least three times. The pH was adjusted to 2.6. The STD NMR experiments were recorded on a Bruker Avance III 500 spectrometer equipped at a temperature of 298 K. The STD-NMR spectra were obtained at a molar ratio of insulin:NK9  = 1∶100 with 3 K scans and selective saturation of insulin resonances either at −0.5 or at 7.3 ppm (40 ppm for reference spectra) using a series of 40 Gaussian-shaped pulses (49 ms, 1 ms delay between pulses), for a total saturation time of 2 s. Subtraction was performed between off and on resonance spectra by phase cycling, leads to the difference spectrum that contains signals arising from the saturation transfer. It is noteworthy to mention that one-dimensional proton NMR spectrum of insulin showed broad lines at low pH (pH 2.6) and the corresponding STD NMR spectrum (on resonance either −0.5 or 7.3 ppm and off resonance 40 ppm) showed very successful saturation transfer across the protein (data not shown). On the other hand, the free peptide, NK9 do not show any STD effects saturating at the same frequency as the complex. Data processing was performed using TOPSPIN program suite.

### Structure prediction of NK9

The starting structure of peptide NK9 for theoretical studies was built using tLeap module of amber. A short minimization was applied for clearing all the steric clashes in the linear starting structure using Amber99SB force field [59]. The coordinates of NK9 were then processed in Desmond MD system for further structure prediction. Simulated annealing (SA) protocol was applied in explicit solvent condition over NK9-peptide for obtaining structural ensembles. The various temperature scales defined in SA are: 10 K–30 ps:100 K–100 ps:300 K–200 ps:400 K–300 ps:400 K–500 ps:300 K–1000 ps. The frames are saved at an interval of 4.8 ps. Python script, “Clustering of Conformers” (Schrödinger, LLC) was used for analysis. The clustering of conformers was done based on the atom-positional root-mean-square difference (RMSDifference).

### Docking studies

Coordinates of insulin heterodimer was obtained from Protein Data Bank (2zp6.pdb). The crystal structure was processed in Protein Preparation Wizard, for rectifying any problem in bond order, overlapping atoms and missing atoms. The water molecules in the crystal structure were kept with an objective that they might show important favorable interactions in subsequent theoretical studies. The macromolecule was further optimized by an iterative process for H-bonding network by re-orienting hydroxyl groups, amide groups of Asn and Gln and choosing appropriate states and orientation of imidazole ring of His residues. The protein was energy minimized before it was processed for docking. Docking protocol as implemented in PatchDock [60] (http://bioinfo3d.cs.tau.ac.il/PatchDock/) was utilized to search possible binding orientation of peptide over insulin. Validation of docked structure was confirmed using STD-NMR. Two interacting models which were in good agreement with STD NMR data were adopted for further MD simulation studies in explicit solvent condition.

### Molecular dynamics (MD) simulation studies

All the simulation studies have been carried out with Amber ff99SB force field in Desmond [61]. The preparation of interacting complex of insulin-NK9 was done at low pH conditions (pH 2.0). Thus, appropriate atomic protonation states were preserved for the micro-canonical system under the force-fields. The macomolecular complex was solvated with 10 Å boundaries from edge in orthorhombic boundary conditions, using TIP3P water models and neutralized with appropriate counter ions [62]. The system was first subjected first to energy minimization with 4000 steps of steepest descent and then to the default relaxation protocol, as present in Desmond MD system. To understand the mechanism of fibrillation delay of insulin by NK9 and to get the structural insight of interactions, MD simulation was performed upto 100 ns time period. NPT ensemble was chosen as micro-canonical system for the biomolecular simulation, with 1 bar pressure and constant temperature of 300 K, controlled using a relaxation time of 1 ps. Electrostatic forces were computed by using Gaussian Split Ewald method [63]. RESPA integrator was used with a 6 fs time step for long-range Coulomb interactions and 2 fs time step for all remaining interactions [64]. M-SHAKE algorithm was used to constraint the bond lengths to hydrogen [65]. The real-space part of the electrostatic and Lennard-Jones interactions was cut off at 10Å. The trajectories were saved at an interval of 5 ps for analysis.

### Principal component analysis (PCA)

Principal component analysis (PCA), which is also known as essential dynamics, is a valuable tool to extract and large-scale correlated atomic motions of a system from simulation trajectory. PCA provides an opportunity to estimate the dominant mode of structural motion and thus the molecular motions can be judged on the basis of either instability or stability. All the data points for PCA analysis were extracted from simulation trajectory using GROMACS program [66]. Trajectory of Desmond simulation was converted into GROMACS format by using VMD. The eigenvector contributed by all atoms; corresponding to collective motion, were extracted using g_anaeig program. The time evolution of collective coordinates for protein backbone (insulin-NK9 complex) during simulation were projected and analyzed.

### Replica exchange molecular simulation (REMD)

We have tried to explore the forms of interactions in the insulin-NK9 complex with the aid of scaling of REMD values for the complex over the period of 8 ns tuning the system temperature. A total of sixteen replicas were run under umbrella sampling as to simulate the insulin-NK9 complex over temperature landscape, viz. 310, 315, 320, 325, 330, 335, 340, 345, 350, 355, 360, 365, 370, 375, 385 and 395 K. The solvation dimensions and ensemble parameters were set similar to the explicit MD simulation studies.

### Aggregation site prediction

The probable prone for the aggregation was predicted using BioLuminate suite [67]. Insulin and complex models from simulation snapshots acquired the appropriate protonation state (pH∼2.0). The parameters for aggregation site prediction were set to 1.0 Å for surface grid spacing, 1.8 Å for probe radius to be used over the van der Waals surface in order to create Connolly surface, 4 Å as cutoff radius for finding hydrophobic neighbors, 1.5 Å^2^ as to specify maximum solvent-accessible surface area for a residue to be regarded as buried.

### Hemolytic assay

Fresh human blood was centrifuged at 4000×g for 10 min and the cell pellet was washed thrice and re-suspended in 10 mM PBS at pH 7.4 to obtain a final concentration of 1×109 erythrocytes/ml. Equal volumes of erythrocytes were incubated with varying concentrations of NK9 peptide with shaking at 37°C for 1 hr. Samples were then subjected to centrifugation at 3500 g for 10 min at 4°C. RBC lysis was measured at different peptide concentrations by taking absorbance at an OD of 540 nm. Complete hemolysis (100%) was determined using 1% Triton X 100 as a control. Hemolytic activity of the NK9 peptide was calculated in percentage using the following Equation 4.
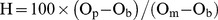
(4)where, O_p_ is the optical density of given peptide concentration, O_b_ is the optical density of buffer and O_m_ is the optical density of Triton X 100.

### *In vitro* cytotoxicity assay

Cell line used in this experiment was HT1080, a human fibro sarcoma cell line obtained from ATCC. Cells were maintained in Dulbecco's modified Eagle's medium (DMEM) containing Penicillin, Streptomycin, Gentamycin and Amphotericin B. For setting up a microwell culture, cells from a frozen stock were spread on a 100 mm dish in DMEM and incubated for 24 hr at 37°C, 5% CO_2_, 100% relative humidity. Cells were then brought into suspension with 0.02% Trypsin, washed with phosphate buffered saline and re-suspended in DMEM. Samples of cell suspension (500 µl) were plated on to 24 well plates at an initial confluency of 30% and grown till it reached 40 to 50%. Varying concentration of peptide was added going up to a maximum concentration of 50 µM and incubated at 37°C, 5% CO_2_ for 44 hr. Cell number was determined by staining cells with addition of 100 µl of methylene blue solution (1% in 50% methanol) to each well followed by incubation at room temperature for 30 min. The stain was aspirated and plates were rinsed with Millipore water twice and air dried. The plates were scanned at 620 nm on a Thermo Scientific Varioskan Flash multimode micro plate reader. An average of 50 absorbance readings taken at different points on each well was measured. Percentage viability of cells was calculated using Equation 5.
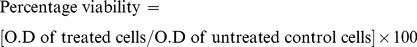
(5)


## Conclusions

In this work, we have developed a nine residue biocompatible peptide, NK9 that is found to interrupt the insulin fibrillation process. Using Thioflavin T fluorescence, we observed that NK9 delays the fibrillation process of insulin in a dose dependent manner. *In situ*, addition of NK9 into the incubating insulin solution decreases the rate of fibrillation process by slowing down the nucleation process, even at a sub-molar ratio. The insulin fibrils formed in presence of NK9 are morphologically different from those formed in its absence. NK9 is found to stabilize native-like secondary structure of insulin thus delaying its dissociation into monomer, which is a precursor for the fibril nucleus. NK9 does interfere with the nucleation process. Fluorescence anisotropy measurement confirms binding of NK9 with insulin with micro-molar affinity and the free energy of binding decreased with increase in temperature indicating hydrophobic nature of the interactions. STD NMR revealed that C^γ^Hs of I2, C^β^Hs of V3, V5 and V8 residues of NK9 are in close proximity to insulin. MD simulation results are in agreement with the binding behavior of insulin with NK9. For the insulin-NK9 complex, aggregation site was found to be Leu^A13^ and Leu^A16^. As per the docked model and simulation trajectory, NK9 was found to be in close proximity of Leu^A13^, Tyr^A14^ and Glu^A17^. Simulation result indicates that only a few hydrophobic residues of NK9 prevent the solvent perturbation influencing insulin fibrillation. Probable fibrillation site of insulin was also identified using BioLuminate software. Such study may provide clue to develop next generation peptides and other organic therapeutics that can be tuned to prevent protein fibrillation.

## Supporting Information

Figure S1 **Spectral assignment (trNOESY spectrum) and sequential walk of NK9 bound to insulin.**
(TIF)

Figure S2 **Prediction of probable aggregation site; using BioLuminate for (A) insulin, (B) insulin-NK9 starting complex, (C) insulin-NK9 complex at 25** **ns, (D) insulin-NK9 complex at 50** **ns, (E) insulin-NK9 complex at 75** **ns, (F) insulin-NK9 complex at 100** **ns.**
(TIF)

Figure S3 **Replica Exchange Molecular Dynamics (REMD) run over insulin-NK9 complex for a time scale of 8ns.** (A and C) RMSD plot for chain A, chain B of insulin and NK9 from 5th replica (330 K) and 6th replica (335 K). (B and D) Temperature variation plots for the trajectories of 5th and 6th replica. (E) Overview of 16 replicas for insulin-NK9 complex which shows the exchange of replicas over the temperature platform in the simulation time course.
(TIF)

Supporting Information S1 **Supporting Information.**
(DOC)

Table S1 **Kinetic parameters of insulin fibrillation.**
(DOC)
