# Supplementary figures and images for: Use of a Small Peptide Fragment as an Inhibitor of Insulin Fibrillation Process: A Study by High and Low Resolution Spectroscopy
Source: PLoS One. 2013 Aug 29;8(8):e72318. doi: 10.1371/journal.pone.0072318 (PMC3756998; doi:10.1371/journal.pone.0072318)

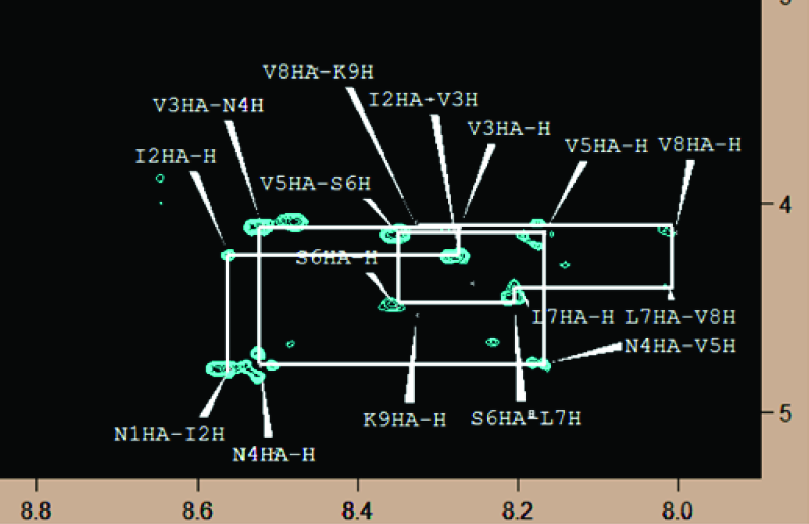

Supplement: Figure S1 — Spectral assignment (trNOESY spectrum) and sequential walk of NK9 bound to insulin. (TIF) [file pone.0072318.s001.tif]

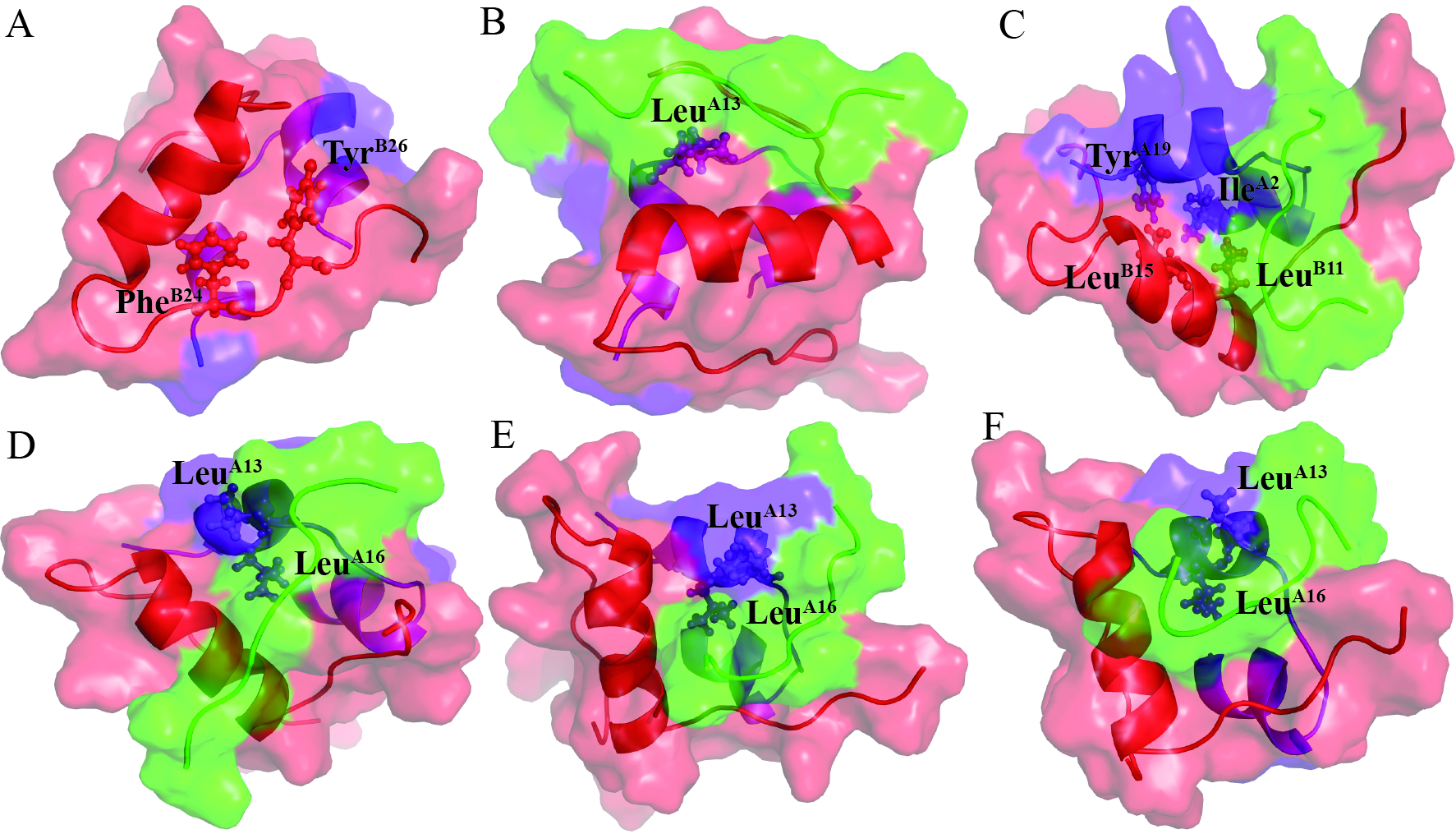

Supplement: Figure S2 — Prediction of probable aggregation site; using BioLuminate for (A) insulin, (B) insulin-NK9 starting complex, (C) insulin-NK9 complex at 25 ns, (D) insulin-NK9 complex at 50 ns, (E) insulin-NK9 complex at 75 ns, (F) insulin-NK9 complex at 100 ns. (TIF) [file pone.0072318.s002.tif]

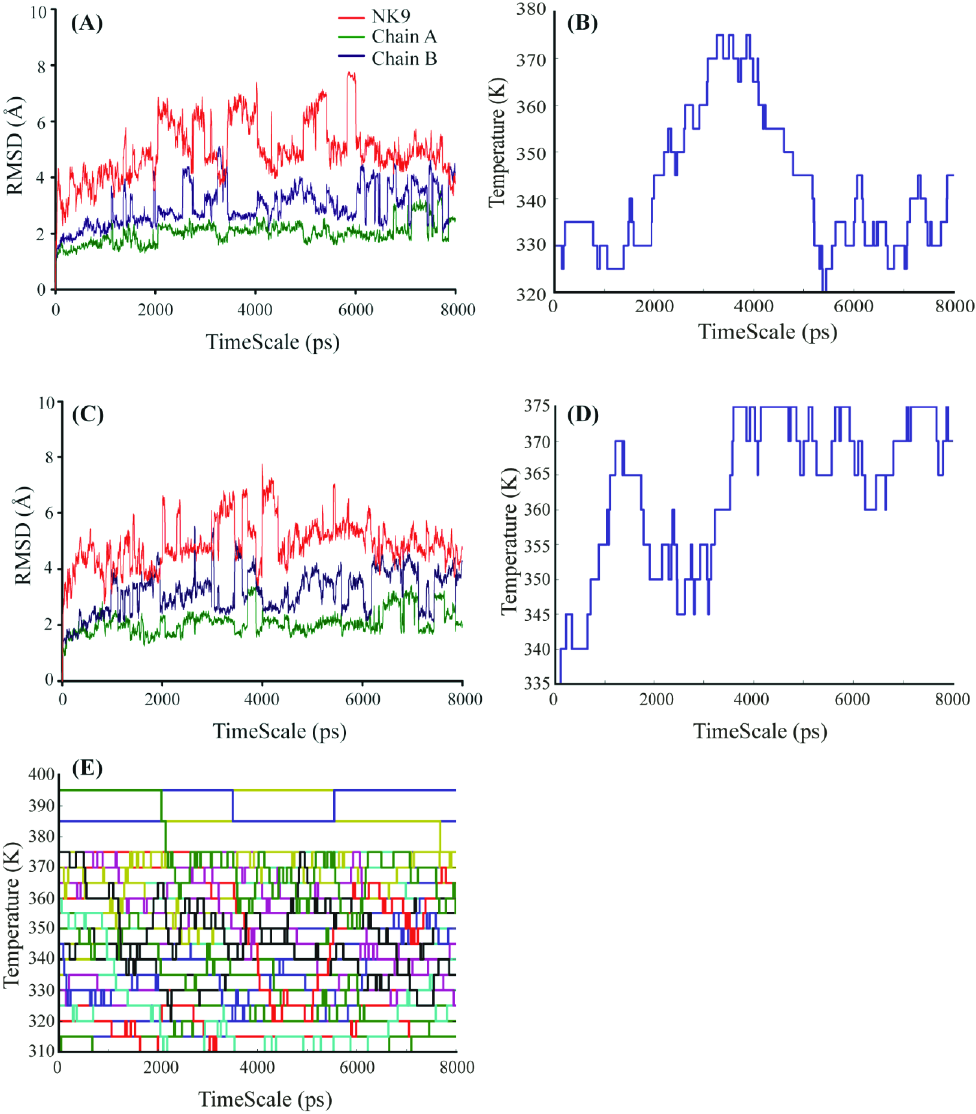

Supplement: Figure S3 — Replica Exchange Molecular Dynamics (REMD) run over insulin-NK9 complex for a time scale of 8ns. (A and C) RMSD plot for chain A, chain B of insulin and NK9 from 5th replica (330 K) and 6th replica (335 K). (B and D) Temperature variation plots for the trajectories of 5th and 6th replica. (E) Overview of 16 replicas for insulin-NK9 complex which shows the exchange of replicas over the temperature platform in the simulation time course. (TIF) [file pone.0072318.s003.tif]
