# Supplementary material for: Use of a Small Peptide Fragment as an Inhibitor of Insulin Fibrillation Process: A Study by High and Low Resolution Spectroscopy
Source: PLoS One. 2013 Aug 29;8(8):e72318. doi: 10.1371/journal.pone.0072318 (PMC3756998; doi:10.1371/journal.pone.0072318)
Supplement: Supporting Information S1 — Supporting Information. (DOC) [file pone.0072318.s004.doc]

**Supporting Information**

Use of a Small Peptide Fragment as an Inhibitor of Insulin Fibrillation Process: A Study by High and Low Resolution Spectroscopy

**Victor Banerjee^1+^, Rajiv K. Kar^2+^, Aritreyee Datta^2^, Krupakar Parthasarathi^3^, Subhrangsu Chatterjee^2^, Kali P. Das^1^*, and Anirban Bhunia^2^***

^1^Department of Chemistry, Bose Institute, 93/1 Acharya Prafulla Chandra Road, Kolkata 700009, India

^2^Biomolecular NMR and Drug Design Laboratory, Department of Biophysics, Bose Institute, Kolkata-700054, India

^3^Department of Microbiology, National University of Singapore, 5 Science Drive 2, Singapore-117597

*Probable site for insulin fibrillation* **–** A probable aggregation site in a macromolecule can be identified based upon the regional hydrophobicity and its solvent accessible surface area. The prediction indicates two residues for insulin macromolecule (Figure S2) namely Phe^B24^ and Tyr^B26^ as prone to initiate aggregation. This is in good correlation with the previous report by Hua et al. [1], which concludes that, the dynamic C-ter of chain B may expose non-polar surfaces. Thereby it enables the aromatic side chains of residues Phe^B24^ – Tyr^B26^ to promote the formation of non-native β-sheet structures. We tried to assess the probable aggregation site in the insulin-NK9 complex using the molecular dynamic simulation where at each 25 ns simulation time the Insulin-NK9 complex was monitored to understand the intermolecular network which boosts the initiation of fibril formation. At the first phase of simulation only Leu^A13^ was determined as the single candidate responsible to initiate the fibrillation but in coarse ongoing simulation four other residues Ile^A2^, Tyr^A19^, Leu^B11^ and Leu^B15^ (Figure S2B and C) were tracked to exhibit their intriguing nature to be the residues which accelerate the fibrillation. This result was manifested from the outlook of the rmsd profile of initial 20 ns simulation time; the rmsd deviation was found very chaotic in nature. It was interesting to find that the residues Leu^A13^ and Leu^A16^ are two possible candidates which can be the key players in dictating the fibrillation process, from the snapshots of simulation the nature of conformation change of these two residues are found static (Figure S2D-F). The visual inspection of the model shows that Leu^A13^ and few of other residues of chain A like Tyr^A14^ and Glu^A17^ were in close proximity of NK9 in the explicit solvent medium. The NK9-insulin complex was bridged with aid of the successful interference of these residues and during the simulation time these residues always show inherent nature to stick to NK9, thus it interprets that without the intervention of these residues the fibrillation process cannot take place. When NK9 is displaced form the surface of interactions of these residues, they are exposed to the solvent as well as the other fibrillar assemblies. Since all the non-polar surface of insulin is not masked by NK9, the solvent perturbation invokes the formation of non-native β-sheet type structures after a certain delay.

1. With the help of cluster analysis, the conformations obtained for NK9 were used for docking with insulin. The docked results were later correlated with the STD-NMR data and only two probable models were used for further study using MD simulation.
2. Sequential assignment of the transferred NOESY spectrum for NK9 in bound state with insulin has been done with SPARKY. The reduced number of inter-residue NOEs of peptide NK9 depicts the unstructured characteristics for NK9 in bound condition.
3. A parallel model (model II) was simulated for a time scale of 100ns with similar conditions as discussed in the main text. Scatter plots for the first three primary eigenvector are shown in Figure S3. Unlike the model I (as described in the main text), model II shows many of the data points wide scattered with respect to 0-50 ns, 50-75 ns and 75 to 100 ns phases of analysis. This indicates that after 100 ns of MD simulation also, the interaction conformation of NK9 with insulin is not favored thermodynamically. With the observation from PCA analysis, it was concluded that model II is not able to provide significant theoretical information and hence discarded in our study.

**Table.**

**Table S1**. Kinetic parameters of insulin fibrillation.

**Figure legends.**

**Figure S1.** Spectral assignment (trNOESY spectrum) and sequential walk of NK9 bound to insulin.

**Figure S2**. Prediction of probable aggregation site; using BioLuminate for (A) insulin, (B) insulin-NK9 starting complex, (C) insulin-NK9 complex at 25 ns, (D) insulin-NK9 complex at 50 ns, (E) insulin-NK9 complex at 75 ns, (F) insulin-NK9 complex at 100 ns.

**Figure S3**. Replica Exchange Molecular Dynamics (REMD) run over insulin-NK9 complex for a time scale of 8ns. (A and C) RMSD plot for chain A, chain B of insulin and NK9 from 5th replica (330K) and 6th replica (335K). (B and D) Temperature variation plots for the trajectories of 5th and 6th replica. (E) Overview of 16 replicas for insulin-NK9 complex which shows the exchange of replicas over the temperature platform in the simulation time course.

**References.**

1. Hua QX, Weiss MA (2004) Mechanism of insulin fibrillation: the structure of insulin under amyloidogenic conditions resembles a protein-folding intermediate. J. Biol. Chem. 279: 21449-21460.
