# Supplementary material for: Use of a Small Peptide Fragment as an Inhibitor of Insulin Fibrillation Process: A Study by High and Low Resolution Spectroscopy
Source: PLoS One. 2013 Aug 29;8(8):e72318. doi: 10.1371/journal.pone.0072318 (PMC3756998; doi:10.1371/journal.pone.0072318)
Supplement: Table S1 — Kinetic parameters of insulin fibrillation. (DOC) [file pone.0072318.s005.doc]

**Table S1**. Kinetic parameters of insulin fibrillation.

| **System** | **Lag Time (min)** | **Apparent Rate**  **Constant (h^-1^)** |
| --- | --- | --- |
| Insulin | 120±5 | 2.9±0.13 |
| Insulin + NK9 50 µM | 147±9 | 2.8±0.12 |
| Insulin + NK9 100 µM | 200±7 | 2.4±0.11 |
| Insulin + NK9 174 µM | 236±11 | 2.6±0.1 |
| Insulin + NK9 350 µM | 246±9 | 3.1±0.16 |
| Insulin + NK9 700 µM | 254±4 | 3.2±0.14 |
